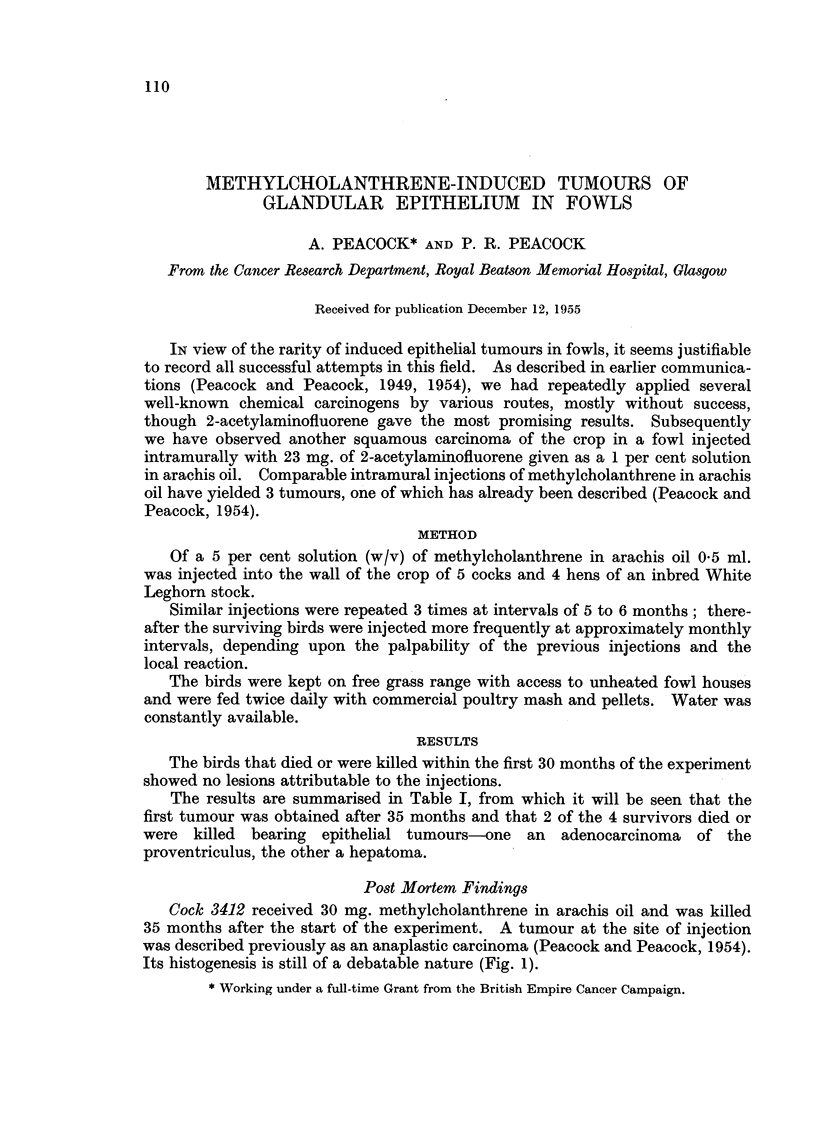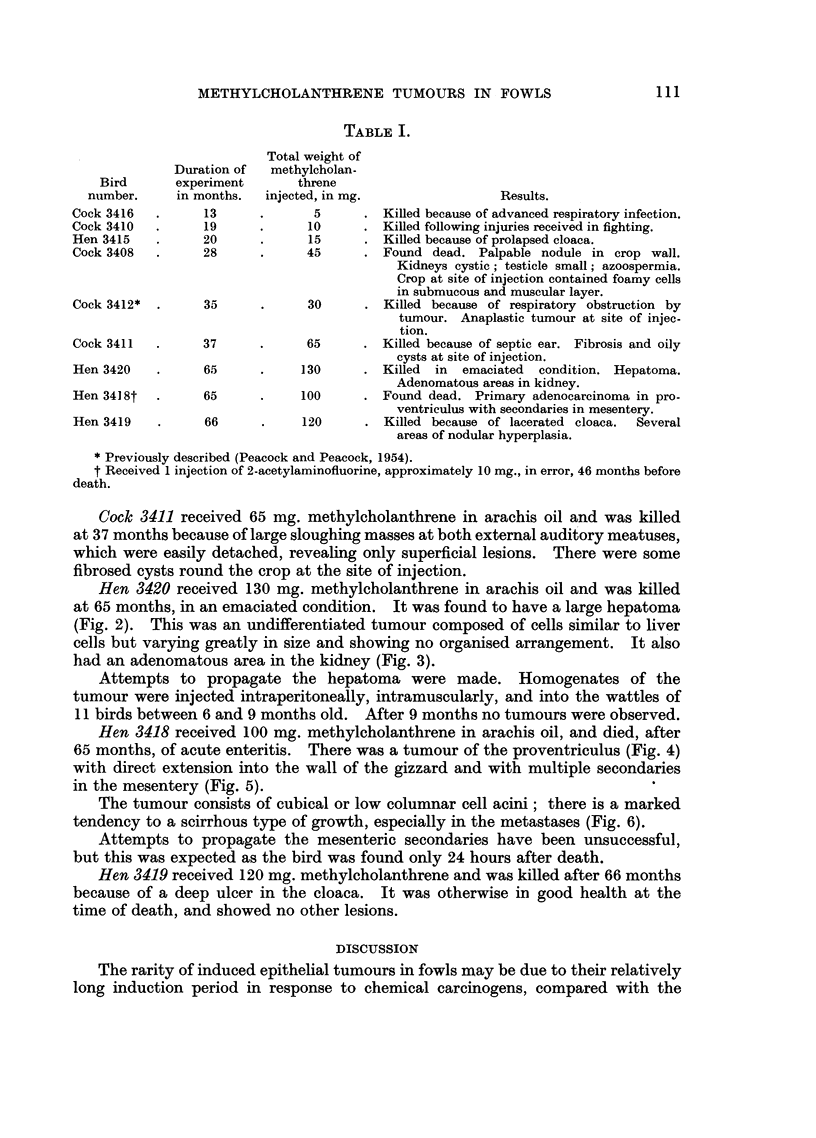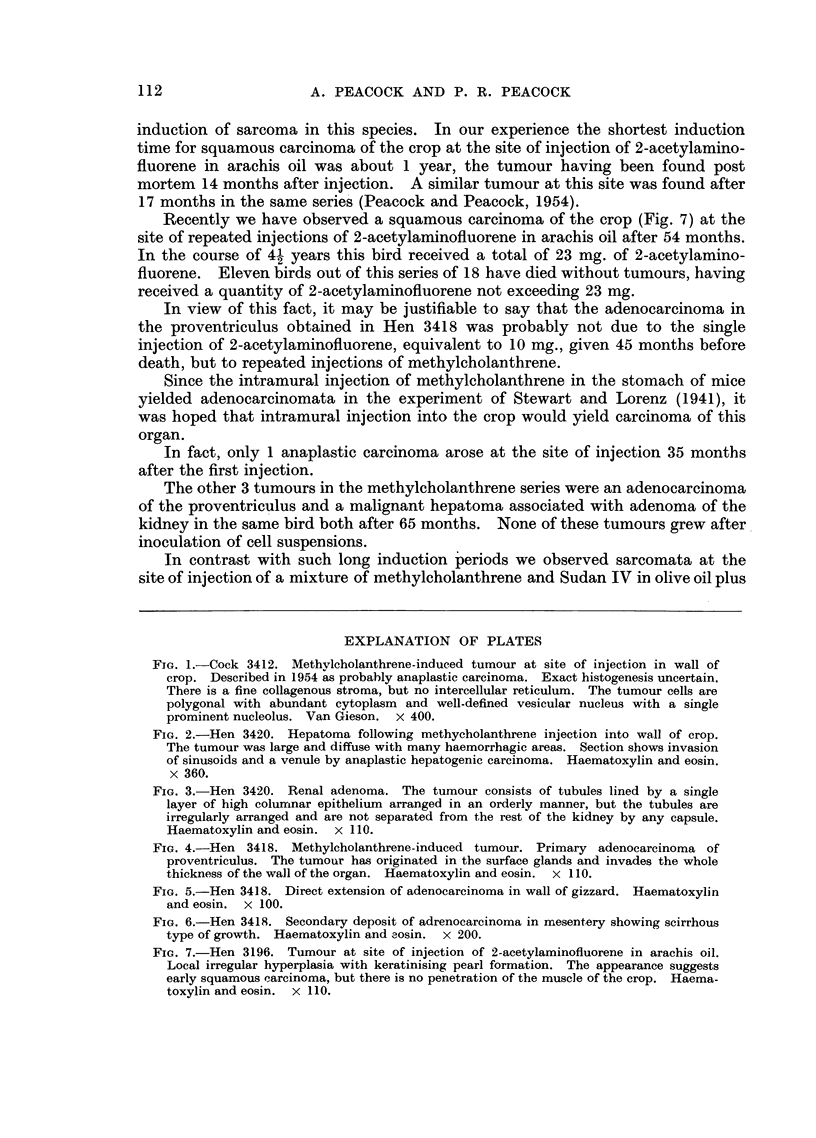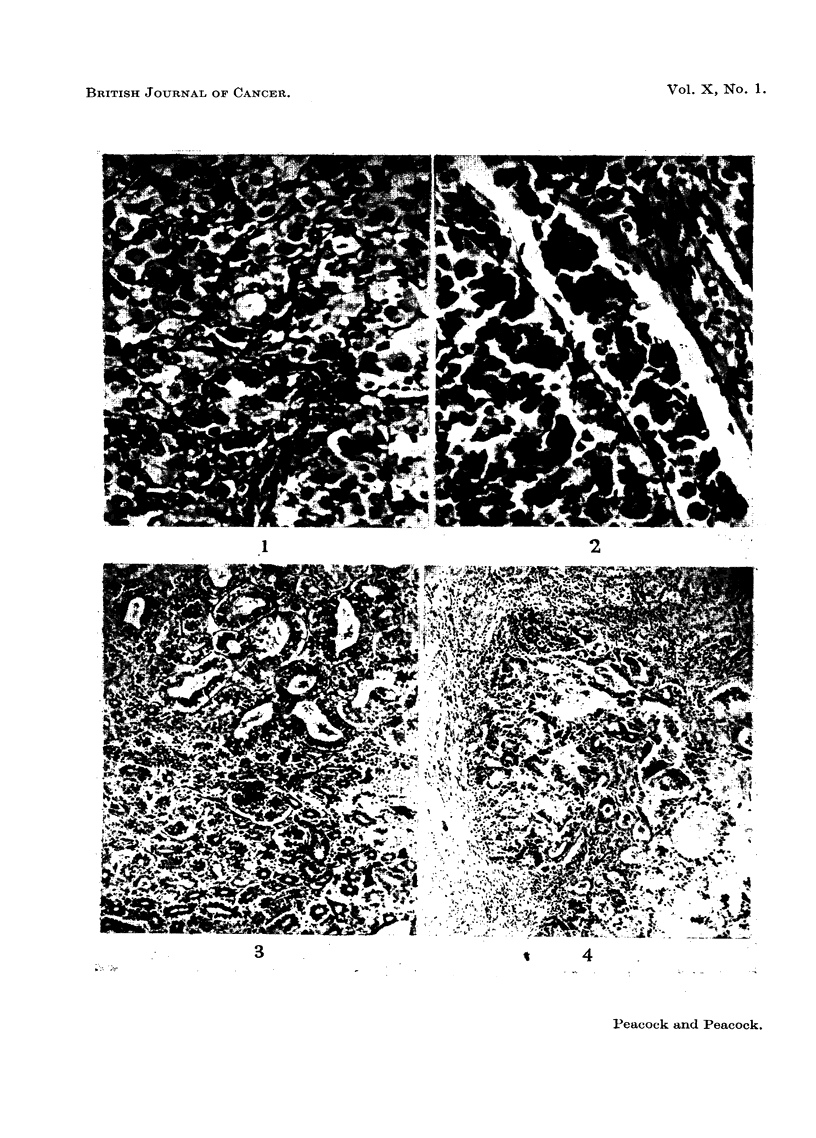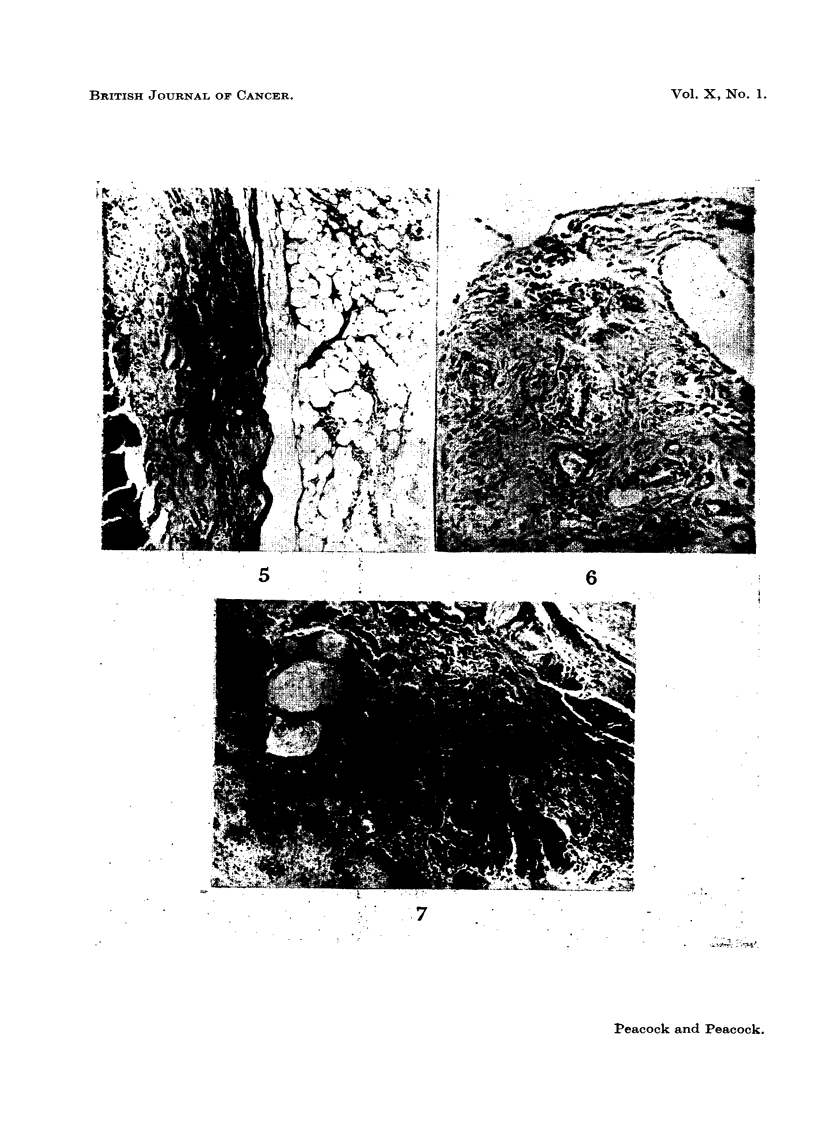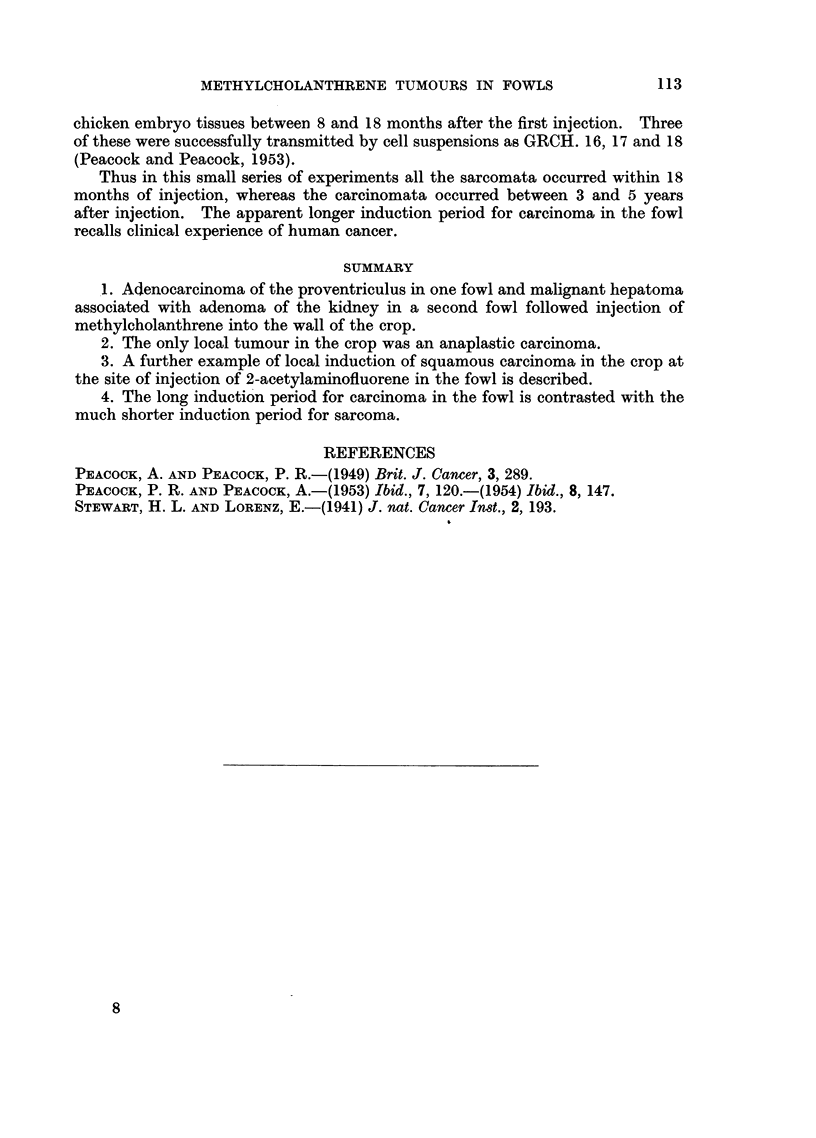# Methylcholanthrene-Induced Tumours of Glandular Epithelium in Fowls

**DOI:** 10.1038/bjc.1956.14

**Published:** 1956-03

**Authors:** A. Peacock, P. R. Peacock

## Abstract

**Images:**


### METHYLCHOLANTHRENE-INDUCED TUMOURS OF GLANDULAR EPITHELIUM IN FOWLS

A. PEACOCK* AND P. R. PEACOCK

*From the Cancer Research Department, Royal Beatson Memorial Hospital, Glasgow*

Received for publication December 12, 1955

IN view of the rarity of induced epithelial tumours in fowls, it seems justifiable to record all successful attempts in this field. As described in earlier communications (Peacock and Peacock, 1949, 1954), we had repeatedly applied several well-known chemical carcinogens by various routes, mostly without success, though 2-acetylaminofluorene gave the most promising results. Subsequently we have observed another squamous carcinoma of the crop in a fowl injected intramurally with 23 mg. of 2-acetylaminofluorene given as a 1 per cent solution in arachis oil. Comparable intramural injections of methylcholanthrene in arachis oil have yielded 3 tumours, one of which has already been described (Peacock and Peacock, 1954).

### METHOD

Of a 5 per cent solution (w/v) of methylcholanthrene in arachis oil 0·5 ml. was injected into the wall of the crop of 5 cocks and 4 hens of an inbred White Leghorn stock.

Similar injections were repeated 3 times at intervals of 5 to 6 months ; thereafter the surviving birds were injected more frequently at approximately monthly intervals, depending upon the palpability of the previous injections and the local reaction.

The birds were kept on free grass range with access to unheated fowl houses and were fed twice daily with commercial poultry mash and pellets. Water was constantly available.

### RESULTS

The birds that died or were killed within the first 30 months of the experiment showed no lesions attributable to the injections.

The results are summarised in Table I, from which it will be seen that the first tumour was obtained after 35 months and that 2 of the 4 survivors died or were killed bearing epithelial tumours—one an adenocarcinoma of the proventriculus, the other a hepatoma.

### *Post Mortem Findings*

*Cock 3412* received 30 mg. methylcholanthrene in arachis oil and was killed 35 months after the start of the experiment. A tumour at the site of injection was described previously as an anaplastic carcinoma (Peacock and Peacock, 1954). Its histogenesis is still of a debatable nature (Fig. 1).

* Working under a full-time Grant from the British Empire Cancer Campaign.

TABLE I.

| Bird number. | Duration of experiment in months. | Total weight of methylcholanthrene injected, in mg. | Results. |
|---|---|---|---|
| Cock 3416 | 13 | 5 | Killed because of advanced respiratory infection. |
| Cock 3410 | 19 | 10 | Killed following injuries received in fighting. |
| Hen 3415 | 20 | 15 | Killed because of prolapsed cloaca. |
| Cock 3408 | 28 | 45 | Found dead. Palpable nodule in crop wall. Kidneys cystic ; testicle small ; azoospermia. Crop at site of injection contained foamy cells in submucous and muscular layer. |
| Cock 3412* | 35 | 30 | Killed because of respiratory obstruction by tumour. Anaplastic tumour at site of injection. |
| Cock 3411 | 37 | 65 | Killed because of septic ear. Fibrosis and oily cysts at site of injection. |
| Hen 3420 | 65 | 130 | Killed in emaciated condition. Hepatoma. Adenomatous areas in kidney. |
| Hen 3418† | 65 | 100 | Found dead. Primary adenocarcinoma in proventriculus with secondaries in mesentery. |
| Hen 3419 | 66 | 120 | Killed because of lacerated cloaca. Several areas of nodular hyperplasia. |

* Previously described (Peacock and Peacock, 1954).
† Received 1 injection of 2-acetylaminofluorine, approximately 10 mg., in error, 46 months before death.

*Cock 3411* received 65 mg. methylcholanthrene in arachis oil and was killed at 37 months because of large sloughing masses at both external auditory meatuses, which were easily detached, revealing only superficial lesions. There were some fibrosed cysts round the crop at the site of injection.

*Hen 3420* received 130 mg. methylcholanthrene in arachis oil and was killed at 65 months, in an emaciated condition. It was found to have a large hepatoma (Fig. 2). This was an undifferentiated tumour composed of cells similar to liver cells but varying greatly in size and showing no organised arrangement. It also had an adenomatous area in the kidney (Fig. 3).

Attempts to propagate the hepatoma were made. Homogenates of the tumour were injected intraperitoneally, intramuscularly, and into the wattles of 11 birds between 6 and 9 months old. After 9 months no tumours were observed.

*Hen 3418* received 100 mg. methylcholanthrene in arachis oil, and died, after 65 months, of acute enteritis. There was a tumour of the proventriculus (Fig. 4) with direct extension into the wall of the gizzard and with multiple secondaries in the mesentery (Fig. 5).

The tumour consists of cubical or low columnar cell acini ; there is a marked tendency to a scirrhous type of growth, especially in the metastases (Fig. 6).

Attempts to propagate the mesenteric secondaries have been unsuccessful, but this was expected as the bird was found only 24 hours after death.

*Hen 3419* received 120 mg. methylcholanthrene and was killed after 66 months because of a deep ulcer in the cloaca. It was otherwise in good health at the time of death, and showed no other lesions.

### DISCUSSION

The rarity of induced epithelial tumours in fowls may be due to their relatively long induction period in response to chemical carcinogens, compared with the induction of sarcoma in this species. In our experience the shortest induction time for squamous carcinoma of the crop at the site of injection of 2-acetylaminofluorene in arachis oil was about 1 year, the tumour having been found post mortem 14 months after injection. A similar tumour at this site was found after 17 months in the same series (Peacock and Peacock, 1954).

Recently we have observed a squamous carcinoma of the crop (Fig. 7) at the site of repeated injections of 2-acetylaminofluorene in arachis oil after 54 months. In the course of $4\frac{1}{2}$ years this bird received a total of 23 mg. of 2-acetylaminofluorene. Eleven birds out of this series of 18 have died without tumours, having received a quantity of 2-acetylaminofluorene not exceeding 23 mg.

In view of this fact, it may be justifiable to say that the adenocarcinoma in the proventriculus obtained in Hen 3418 was probably not due to the single injection of 2-acetylaminofluorene, equivalent to 10 mg., given 45 months before death, but to repeated injections of methylcholanthrene.

Since the intramural injection of methylcholanthrene in the stomach of mice yielded adenocarcinomata in the experiment of Stewart and Lorenz (1941), it was hoped that intramural injection into the crop would yield carcinoma of this organ.

In fact, only 1 anaplastic carcinoma arose at the site of injection 35 months after the first injection.

The other 3 tumours in the methylcholanthrene series were an adenocarcinoma of the proventriculus and a malignant hepatoma associated with adenoma of the kidney in the same bird both after 65 months. None of these tumours grew after inoculation of cell suspensions.

In contrast with such long induction periods we observed sarcomata at the site of injection of a mixture of methylcholanthrene and Sudan IV in olive oil plus

---

### EXPLANATION OF PLATES

Fig. 1.—Cock 3412. Methylcholanthrene-induced tumour at site of injection in wall of crop. Described in 1954 as probably anaplastic carcinoma. Exact histogenesis uncertain. There is a fine collagenous stroma, but no intercellular reticulum. The tumour cells are polygonal with abundant cytoplasm and well-defined vesicular nucleus with a single prominent nucleolus. Van Gieson. × 400.

Fig. 2.—Hen 3420. Hepatoma following methycholanthrene injection into wall of crop. The tumour was large and diffuse with many haemorrhagic areas. Section shows invasion of sinusoids and a venule by anaplastic hepatogenic carcinoma. Haematoxylin and eosin. × 360.

Fig. 3.—Hen 3420. Renal adenoma. The tumour consists of tubules lined by a single layer of high columnar epithelium arranged in an orderly manner, but the tubules are irregularly arranged and are not separated from the rest of the kidney by any capsule. Haematoxylin and eosin. × 110.

Fig. 4.—Hen 3418. Methylcholanthrene-induced tumour. Primary adenocarcinoma of proventriculus. The tumour has originated in the surface glands and invades the whole thickness of the wall of the organ. Haematoxylin and eosin. × 110.

Fig. 5.—Hen 3418. Direct extension of adenocarcinoma in wall of gizzard. Haematoxylin and eosin. × 100.

Fig. 6.—Hen 3418. Secondary deposit of adrenocarcinoma in mesentery showing scirrhous type of growth. Haematoxylin and eosin. × 200.

Fig. 7.—Hen 3196. Tumour at site of injection of 2-acetylaminofluorene in arachis oil. Local irregular hyperplasia with keratinising pearl formation. The appearance suggests early squamous carcinoma, but there is no penetration of the muscle of the crop. Haematoxylin and eosin. × 110.

1

2

3

4

Peacock and Peacock.

5

6

7

Peacock and Peacock.

chicken embryo tissues between 8 and 18 months after the first injection. Three of these were successfully transmitted by cell suspensions as GRCH. 16, 17 and 18 (Peacock and Peacock, 1953).

Thus in this small series of experiments all the sarcomata occurred within 18 months of injection, whereas the carcinomata occurred between 3 and 5 years after injection. The apparent longer induction period for carcinoma in the fowl recalls clinical experience of human cancer.

### SUMMARY

1. Adenocarcinoma of the proventriculus in one fowl and malignant hepatoma associated with adenoma of the kidney in a second fowl followed injection of methylcholanthrene into the wall of the crop.

2. The only local tumour in the crop was an anaplastic carcinoma.

3. A further example of local induction of squamous carcinoma in the crop at the site of injection of 2-acetylaminofluorene in the fowl is described.

4. The long induction period for carcinoma in the fowl is contrasted with the much shorter induction period for sarcoma.

### REFERENCES

Peacock, A. and Peacock, P. R.—(1949) *Brit. J. Cancer*, **3**, 289.
Peacock, P. R. and Peacock, A.—(1953) *Ibid.*, **7**, 120.—(1954) *Ibid.*, **8**, 147.
Stewart, H. L. and Lorenz, E.—(1941) *J. nat. Cancer Inst.*, **2**, 193.